# Mediators in the Relationship between Internet Addiction and Body Mass Index: A Path Model Approach Using Partial Least Square

**Published:** 2018-08-18

**Authors:** Hamid Reza Tabatabaee, Abbas Rezaianzadeh, Mehdi Jamshidi

**Affiliations:** ^1^ Research Center for Health Sciences, Department of Epidemiology, School of Health, Shiraz University of Medical Sciences, Shiraz, Iran; ^2^ Department of Epidemiology, Faculty of Public Health, Shiraz University of Medical Sciences, Shiraz, Iran; ^3^ Colorectal Research Center, Faghihi Hospital, Shiraz University of Medical Sciences, Shiraz, Iran; ^4^ Student Research Committee, Department of Epidemiology, School of Health, Shiraz University of Medical Sciences, Shiraz, Iran; ^5^ Behbahan Faculty of Medical Sciences, Behbahan, Iran

**Keywords:** Body Mass Index, Internet Addiction, Adolescents, Iran

## Abstract

**Background:** Adolescence obesity has now become an epidemic. In recent years, Internet addiction has been identified as a risk factor for obesity. We aimed to evaluate the role of some mediators such as sleep quality, physical activity and fast food consumption in the relation between internet addiction and Body Mass Index (BMI) among adolescents.

**Study design:** A cross-sectional study.

**Methods:** Overall, 928 students, aged between 13 and 17 yr, were randomly selected in Behbahan, southwestern Iran from Oct 2017 to Dec 2017. Data were collected using a demographic survey, Young's internet addiction, Pittsburgh sleep quality, and food frequency, questionnaires. Data analysis was performed using Partial Least Squares (PLS) path analysis.

**Results:** PLS path analysis revealed that the direct effect of Internet addiction on BMI was (Path Coefficient = 0.16, [95% CI: 0.12- 0.21]). Moreover, the indirect effect of internet addiction on BMI through sleep quality was (f^2^ = 0.12 (*P*<0.001)), physical activity (f^2^ =0.04 (*P*<0.001)), and fast food consumption ( f^2^ = 0.05 (*P*<0.001)).

**Conclusions:** Results of this study regarding the relationship between internet addiction and BMI and the effect of this phenomenon on sleep quality, physical activity and dietary habits suggest planning prevention and treatment programs to reduce the prevalence of this phenomenon in schools.

## Introduction

Adolescents’ obesity has been introduced by WHO as an epidemic in the 21st century^1^. Body Mass Index (BMI) at or above 85^th^and 95^th^ percentiles are defined as overweight and obesity for adolescents, respectively^2-4^.

Overweight and obesity are associated with increased blood pressure, diabetes,‏ and other non-communicable diseases in adolescents^2,5,6.^ In addition, many psychosocial effects result including low self-confidence, social isolation, drug abuse, anxiety, depression and even suicide^2^.

About 1.2 million adolescents worldwide are overweight and obese, among them, 88% are living in developing nations^7^. The prevalence of obesity in Iranian adolescents increased from 10.4% to 10.8% between 2005 and 2010 and was shown to have an increasing trend in recent years.

Due to the increasing the prevalence of obesity among adolescents in recent years, novel environmental risk factors such as internet addiction have been studied as an independent risk factor associated with obesity^8-11^.

Although a standardized definition has not‏ been uniformly agreed upon, Internet addiction involves an individual’s inability to control‏ his or her use of the Internet, negative consequences (e.g., failing in school, decreased productivity), and marked distress‏ and functional impairment^12,13^. People with internet addiction neglect not only their responsibilities and obligations at home and society but also refuse to do physical activity^10^.

Numerous studies have been conducted on the relationship between Internet addiction and BMI in school-aged adolescents ^8-10,14^. In China, the prevalence of internet addiction was significantly higher in overweight and obese people^11^. The direct relationship has been widely examined, while there is no clear biological mechanism for the direct relationship, as internet addiction is associated with the BMI without any mediator.

The main effect of this relationship is mediated by other factors. Internet addiction can reduce the duration of physical activity, the duration, and quality of sleep, and lead to use unhealthy food products ^15^ which, can finally lead to overweight and obesity in adolescents.

In Iran, most Internet addiction studies focused on the prevalence or psychological features, and the association between internet addiction and BMI in adolescents has been less studied. In a few studies conducted in Iran, the prevalence of internet addiction varied from 15% to 22% ^16,17^. No similar studies have been carried out on the prevalence of internet addiction and its relation with BMI in the presence of the mediator variables among adolescents in Iran.

Therefore, we aimed to estimate the prevalence of internet addiction in the adolescents aged 7-17 yr in Behbahan City, and examine important pathways through which internet addiction can associate with obesity using path analysis.

## Methods

### Study design

In this cross-sectional study, 928 students, aged between 13 and 17 yr, were selected in Behbahan City, southwestern Iran between Oct 2017 and Dec 2017.

### Study population

Sample size calculation was based on prevalence of overweight in adolescents 16.5% based on a previous study^18^, a precision of 3% and confidence level of 95%, considering the design effect 1.5 and 5% of non-response, the required sample size was calculated to be 928.

Subjects were selected using multistage sampling. At first stage, the population was divided into urban and rural area (strata), and a total of four health centers were selected as cluster from each area (clusters). Then, a number of schools were selected from each cluster. Eventually, a class in each school was randomly selected. All students of the selected classes were recruited into the study. The questionnaires were distributed after the oral consent was obtained from all participants.

The inclusion criteria were students aged 13-17 yr. The exclusion criteria included students with asthma, hypertension, diabetes, heart diseases, and sleep apnea

Informed consent was obtained from the parents of students for participation.

### Internet addiction

The Internet addition was evaluated using Young's internet addiction questionnaire, widely used in international studies^10^, and its validity and reliability have been proved in previous studies,^20^. This questionnaire consists of twenty items with Likert scale ranging from 1 to 5, and the total score for each individual will be between 20 and 100. A score of 20 to 49 shows an average online user, the scores between 50 and 69 represent occasional or frequent problems due to internet, and score greater than 70 is classified as significant problems^10^. This questionnaire is comprised of six psychological components including salience, excessive use, anticipation, neglect of work, neglect of social life, and lack of control^20^. The Iranian version of this questionnaire was shown to have acceptable reliability and validity^21^ with a Cronbach’s alpha of 0.93. The Cronbach’s alpha of the present study was calculated 0.86.

### Sleep quality

Sleep quality was also assessed using a standard and valid questionnaire of Pittsburgh sleep quality index^22^ which included seven components, subjective sleep quality, sleep latency, sleep duration, habitual sleep efficiency, sleep disturbances, use of sleeping medication, and daytime dysfunctions. The items in this questionnaire are based on a 4-point Likert scale. The score of each item is from ranged from 0 to 3. The final score of the questionnaire will be the total score of seven components, which will be 0 to 21 for each person. A score over 5 will represent poor sleep quality, and the score of less than 5 indicate good sleep quality. The Iranian version of this questionnaire showed acceptable validity and reliability^23^ with a Cronbach’s alpha of 0.78. The reliability of this study was calculated (Cronbach’s alpha=0.74).

### Dietary habits

We evaluated dietary habits using the food frequency questionnaire (FFQ), comprised of 87 items on all major food groups and other types of food. The consumption of each food was defined as "the frequency of consumption per day", "the frequency of consumption per week", "the frequency of consumption per month", and "do not consume". The frequencies of consumption of all types of food were converted into daily frequencies and then, data were entered into the statistical software. This questionnaire revealed a good validity and reliability in similar studies in the studied age group (Pearson correlation coefficients between test and retest for foods was r=0.82) ^24^‏. The reliability of the questionnaire in this study was (Cronbach’s alpha=0.80).

Fast food consumption of each subject was extracted using the information obtained from the questionnaire.

In order to determine the physical activity, participants were asked about the number of days and hours they do physical activity per week, and finally, the physical activity of each person was determined in average minutes per day.

### Anthropometric measurements

Weight was measured and recorded in kilogram with standard and portable scales with minimal error, with light clothes and no shoes. Height was measured and recorded to the nearest millimeter without shoes, using a portable stadiometer. BMI was also calculated as weight in kilograms divided by height in meters squared.

Finally, the BMI percentile was calculated by matching the BMI with age and sex, based on the Centers for Disease Control and Prevention (CDC) growth charts^25^.

According to the WHO Expert Committee, individuals based on the BMI percentile were categorized into four groups: underweight (less than or equal to the 5th percentile), normal or healthy weight (between the 5th and 85th percentile), overweight (between the 85th to 95th percentile), and obese (over 95th percentile)^3^.

### Statistical analysis

Data analysis was done with SPSS software ver. 20 (Chicago, IL, USA) for descriptive analysis and path analysis was performed using Smart PLS-3^26^. Statistical significance was determined in 0.05 level.

Partial least squares method (PLS) is a variance-based method, in contrast to older methods based on covariance. This method usually is used when the model theory has not been developed sufficiently, as well as when the sample size is small or there are non-normal data, so there would be no specific presumption about the data distribution in this method^18^.

To evaluate the structural model in PLS, the coefficient of determination‏) R^2^) is used and, the values ​​of 0.25, 0.50 and 0.75 are considered as weak, moderate and strong, respectively. The effect size of the variables (f^2^) in the model also represents the extent to which each independent variable affects the dependent variable, with the values ​​of 0.02, 0.15, 0.35 classified as weak, moderate and strong, respectively. In the PLS model, the minimum value of 0.7 is considered for factor loadings^18^.

The hypothetical model of this study was designed based on the relationships examined in the previous studies ^27-39^. However, there is no study that determined the mediating effects on internet addiction and BMI.

**Figure 1:**
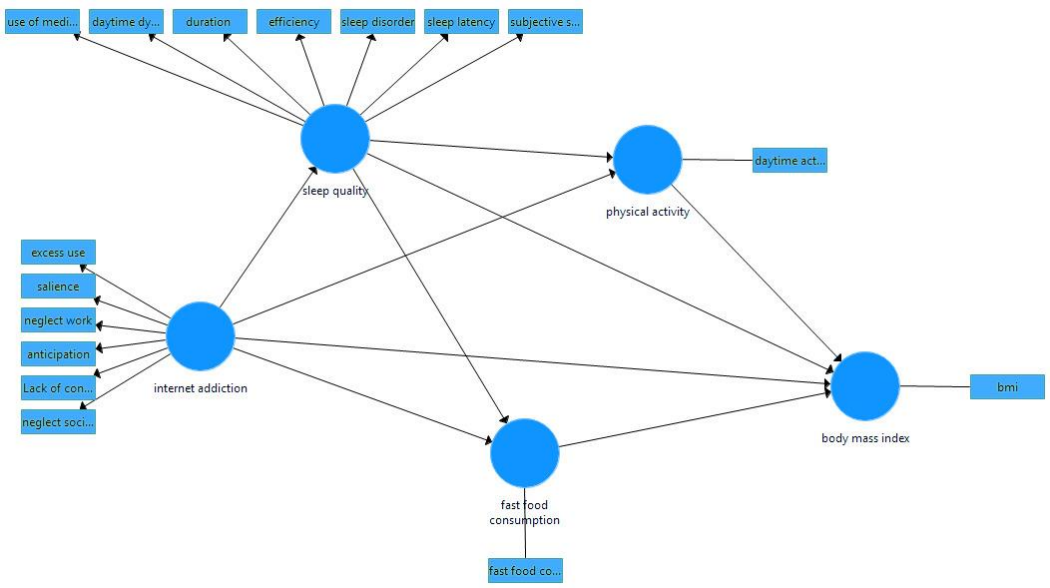
The hypothesized model of the relationship between internet addiction, the sleep quality, physical activity, and fast food consumption with BMI among students

The hypothesized model was designed according to Figure 1. The existence of non-normal data and non-development of the hypothesized model are the main reasons for path analysis using the Partial least square method.

## Results

This study was conducted on 904 students. Overall, 24 subjects were excluded from the study. The mean and standard deviation of the participants' age in the study was 15.03 ± 1.34 year.‏

Table 1 displays the sample characteristics of participating adolescents in the total sample stratified‏ according to gender. About 49% of the participants were male and 51% were female. 77.1% of the subjects were from urban area and 22.9 were from rural area.

**Table 1:** Sample characteristics of participating adolescents according to gender with absolute numbers and percent of categorical variables

**Variables**	**Male, n=443**		**Female, n=461**		**Total**	
	**Number**	**Percent**	**Number**	**Percent**	**Number**	**Percent**
Residence						
City	341	76.9	356	77.2	697	77.1
Rural	102	23.1	105	22.8	207	22.9
Family size					
≤4	228	51.5	216	46.9	444	49.1
5-6	194	43.8	218	47.3	412	45.6
7-9	20	4.5	27	5.8	47	5.2
≥10	1	0.2	0	0.0	1	0.1
Father's education					
Illiterate	9	2.0	18	3.9	27	3.0
Primary school	45	10.1	43	9.3	88	9.8
Middle school	69	15.6	101	21.9	170	18.8
High school	126	28.5	113	24.5	239	26.4
Academic	194	43.8	186	40.4	380	42.0
Mother's education					
Illiterate	7	1.6	12	2.6	19	2.1
Primary school	71	16.0	72	15.6	143	15.9
Middle school	103	23.3	108	23.4	211	23.3
High school	122	27.5	132	28.6	254	28.1
Academic	140	31.6	137	29.8	277	30.6
Type of school					
public	352	79.5	378	82.0	730	80.8
private	91	20.5	83	18.0	174	19.2
Computer availability in bedroom			
yes	168	37.9	171	62.9	339	37.5
no	275	62.1	290	37.1	565	62.5

The prevalence of Internet addiction (IATEST ≥50) in this study was 12.71%, and poor sleep quality (PSQI SCORE >5) was 21.12%. Moreover, the prevalence of overweight and obesity in the study population were 17.69% and 6.19%, respectively (Table 2).

**Table 2:** Characteristics of participants (for variables‏ in the model) by gender

**Variables**	**Male, n=443**		**Female, n=461**		**Total**	
	**Number**	**Percent**	**Number**	**Percent**	**Number**	**Percent**
BMI (kg/m^2^)					
Underweight	20	4.5	14	3.0	34	3.8
Normal	311	70.2	343	74.4	654	72.3
Overweight	83	18.7	77	16.7	160	17.7
Obese	29	6.6	27	5.9	56	6.2
Internet addiction (score)				
≤50	390	88.0	399	86.6	789	87.3
50-69	43	9.7	51	11.1	94	10.4
>70	10	2.3	11	2.4	21	2.3
Sleep quality (score)				
<5	353	79.7	360	78.1	713	78.9
≥5	90	20.3	101	21.9	191	21.1

The mean and standard deviation of daily physical activity in minute, and frequency of fast food consumption per week were 66.97±33.5 and 2.08±1.53, respectively.

The prevalence of internet addiction within BMI‏ percentile categories stratified by gender is shown in Table 3. 22.32% and 25.32% of the male and female at 85th percentile or higher displayed some degree of internet addiction respectively.

**Table 3:** The prevalence of internet addiction among adolescents within BMI groups‏ by gender

**Internet addiction (score)**	**Male, n=443**				**Female, n=461**			
	**Underweight**	**Normal**	**Overweight**	**Obese**	**Underweight**	**Normal**	**Overweight**	**Obese**
≤50	18	285	67	20	13	310	58	18
50-69	2	23	10	8	1	29	14	7
>70	0	3	6	1	0	4	5	2

**Figure 2:**
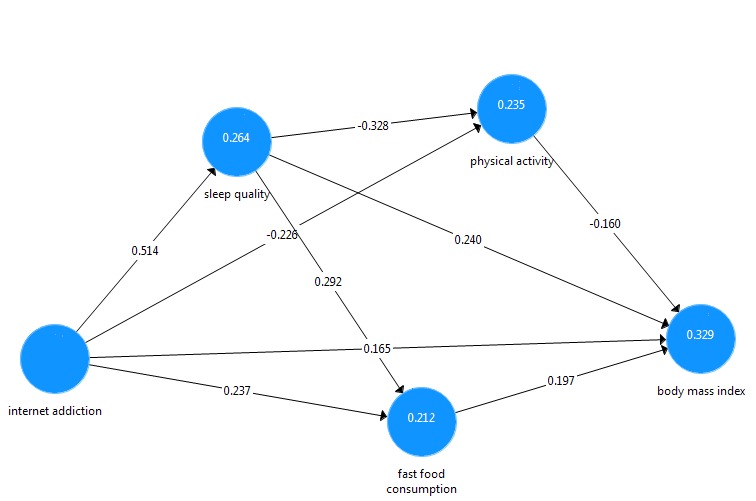
Path diagram for the partial least squares (PLS) model linking relationship internet addiction, sleep quality, physical activity and fast food consumption with BMI.

After testing the model, considering the low factor loading (0.16), the component, use of medication was eliminated from the model due to the low consumption of sleep medication in the study population, and limited number of items on sleep quality, in order to achieve better fitting ^18^. Since component of salience was collinear with the other components of internet addiction and it has the same concept as excessive use, it was integrated into this component. The final model is presented in Figure 2. The validity and reliability of the structural model in PLS include the Cronbach's Alpha, the Average Variance Extracted (AVE), Composite Reliability and the rho-A, and the values for all constructs show the appropriate validity and reliability of the model.

The model fitting of the path analysis in PLS3 was evaluated by Standardized Root Mean Square Residual (SRMR‏(, Normed Fit Index (NFI), Exact Model Fit, Chi², and Rms theta . As such, the values of SRMR <0.08 and NFI> 0.9 represent a good model fitting, other fit indicators of the model are also acceptable.

The R^2^values for the endogenous variables of the model, sleep quality, fast food, and physical activity were calculated at 0.26, 0.21, and 0.23, respectively. Moreover, according to the results of the path analysis, 32% of the variance of BMI of the study population is explained by internet addiction, sleep quality, fast food consumption, and physical activity. Table 4 shows direct, indirect and total effects of exogenous variables on the endogenous variables. The indirect effects of internet addiction were shown to be more than direct effects, indicating the effect of mediating variables in the model.

**Table 4:** The direct, indirect and total effects of independent variables on BMI

**Latent variable**	**Direct effect**	**Indirect effect**	**Total effect**
Internet addiction	0.16	0.26	0.42
Sleep quality	0.24	0.11	0.35
physical activity	-0.16	0.00	-0.16
Fastfood consumption	0.19	0.00	0.19

The effect size (f^2^) of internet addiction on fast food consumption, physical activity, and BMI were 0.05, 0.02, and 0.04, respectively showing the medium effect size of internet addiction on these three variables. The effect size of internet addiction on sleep quality was 0.35, reflecting the large effect size (f^2^) of this variable on sleep quality. The effect size (f^2^) of sleep quality on physical activity, fast food consumption and BMI was 0.1, 0.08 and 0.05, respectively, indicating a moderate effect size (f^2^). Similarly, the effect size (f^2^) of physical activity and fast food consumption on BMI were 0.02 and 0.04 respectively, which represents the medium effect size (f^2^) in the model.

Table 5 shows the path coefficients in the final model. Results of table three demonstrate that all paths are significant at a level of *P*<0.001, with the path of internet addiction to the sleep quality to obesity as the highest path coefficient leading to the BMI in the model (f^2^= 0.12, *P*<0.001).

**Table 5:** Path coefficients in the original sample and the mean of the bootstrapped samples‏ with bootstrapped t-test estimates and *P* values for BMI

**Path**	**Original Sample**	**Sample Mean**	**SD**	**t-test**	***P*** ** value**
Internet addiction to fast food consumption to BMI	0.047	0.047	0.011	4.241	0.001
Internet addiction to sleep quality to fast food consumption to BMI	0.03	0.03	0.006	4.555	0.001
Internet addiction to physical activity to BMI	0.036	0.036	0.009	4.145	0.001
Internet addiction to sleep quality to physical activity to BMI	0.027	0.027	0.006	4.726	0.001
Internet addiction to sleep quality to BMI	0.123	0.124	0.018	7.015	0.001
Internet addiction to sleep quality to fast food consumption	0.15	0.15	0.019	7.724	0.001
Internet addiction to sleep quality to physical activity	-0.169	-0.169	0.018	9.153	0.001

## Discussion

The aim of this study was to determine the prevalence of internet addiction in adolescents and to investigate the relationship between internet addiction and BMI and the mediating role of sleep quality, physical activity, and fast food consumption in adolescents. The prevalence of Internet addiction in this study was 12.7%. In Iran, the prevalence of internet addiction was reported as 22.2% among students ^16^.

The difference between the results of this study with our findings may be due to age differences in the study subjects, as this study investigated high school students, whereas we included middle school and high school students. Besides, only students from urban areas were recruited, while we recruited students from both urban and rural areas. In another study, the prevalence of Internet addiction was 15% among adolescents aged 14-16 yr^17^, which was in compliance with the findings of our study. In China, the prevalence of Internet addiction among Chinese adolescents was 21.23 ^11^.

Findings of the present study showed that 24.5% of the overweight subjects showed some degree of internet addiction, while in non-overweight individuals, the prevalence of internet addiction was 10%. The study on Turkish adolescents supported these findings. In addition, there was a significant relationship between internet addiction and BMI in Turkish students aged 14-18 yr^10.^

In the present study, the indirect effects of Internet addiction on BMI can be explained by many factors. There was a significant positive relationship between Internet addiction and sleep quality, which was in accordance with previous studies, poor sleep quality is more prevalent among internet addict. Internet addiction reduces the quality of sleep^40^. Internet addiction was associated with poor sleep quality ^27,28^. Moreover, Internet addiction was associated with decreased tendency for physical activity ^34,35^, impaired hormonal balance, increased ghrelin level and reduced level of Leptin hormone can increase feeling of hunger and eventually increase calorie intake, leading to overweight and obesity^41^. Poor sleep quality as one of the consequences of Internet addiction can also increase the tendency to use unhealthy foods. 15.8% of the people who reported good sleep quality, were fast food consumers, whereas 19.5 % with poor sleep quality reported fast-food consumption^32^. In addition, there was a significant inverse relationship between internet addiction and physical activity. Internet addiction through reduction of physical activity leads to an increased risk of overweight and obesity ^29^. Physical activity in people with no internet addiction was 55%, while it was reported 47% among internet addicts. Students with signs of internet addiction reported unhealthier lifestyle and consumed more fast foods. In this study, 20% of people with Internet addiction used fast food daily, while 16% without internet addiction reported using fast foods^31^. All paths were significant in the final model and found that internet addiction is, directly and indirectly, related to the BMI. Generally, the effect size of the model variables was shown to be small and medium. The effect size of the variables in this model is attributed to the prevalence of the study variable. Relatively intermediate prevalence of variables in the model has made the coefficients of correlation between the variables of the model low and ultimately leads to the small and medium effect sizes in the model. In similar populations in which the prevalence of Internet addiction, poor sleep quality, insufficient physical activity and fast food consumption is higher, these variables will reveal greater effect size in the similar model.

This is the first study that examined the role of variables mediated in the relationship between Internet addiction and the BMI by using path analysis. It is also the first study in Iran that investigated the relationship between Internet addiction and BMI among children and adolescents. This is a cross-sectional study that makes causality investigation impossible. Moreover, the precision of self-reporting questionnaire may not be sufficient for measuring some variables, such as sleep quality and dietary habits. Otherwise, lack of measurement of other latent variables, such as personal psychosocial characteristics including depression, anxiety, etc., that can mediate this relationship, and the direct relationship found between internet addiction and BMI can be due to the absence of these variables. Eventually, other variables can be effective in parallel with internet addiction in the above relationships, including watching television and playing computer video games, but it was impossible to measure all these variables in this study.

## Conclusion

According to the results of this study and the confirmation of the relationship between Internet addiction and BMI and the effect of this phenomenon on sleep quality, physical activity, and dietary habits, planning prevention and treatment programs to reduce the prevalence of this phenomenon in schools is of great importance.

## Acknowledgements

The present paper was extracted from the M.Sc. thesis in Epidemiology written by Mehdi Jamshidi and financially supported by Shiraz University of Medical Sciences (grant No. 95.01.04.13225). Hereby, the authors would like to thank management board of Behbahan Education. They would also like to express their gratitude to all study participants.

## Conflict of interest statement

The authors declare no conflict of interest.

## Funding

The present article was financially supported by Shiraz University of Medical Sciences (grant No. 95.01.04.13225).

## Highlights

- The prevalence of Internet addiction in this study was 12.7%.
- The Internet addiction has been higher in overweight and obese adolescents.
- Path analysis revealed that Internet addiction, both directly and indirectly, was associated with Body Mass Index.
- Prevention programs are needed to reduce the prevalence of Internet addiction.
